# Supplementary material for: Seroprevalence of HIV, HBV, and syphilis co-infections and associated factors among pregnant women attending antenatal care in Amhara regional state, northern Ethiopia: A hospital-based cross-sectional study
Source: PLoS One. 2024 Aug 8;19(8):e0308634. doi: 10.1371/journal.pone.0308634 (PMC11309376; doi:10.1371/journal.pone.0308634)
Supplement: Attachment — Submitted filename: response to reviewers.docx [file pone.0308634.s002.docx]

To: The Academic Editor, PLOS ONE

Re: Manuscript PONE-D-24-25429

'Seroprevalence of HIV, HBV, and Syphilis Co-infections and Its Associated Factors Among Pregnant Women Attending Antenatal Care in Amhara Regional State, Northern Ethiopia'

Dear Academic Editor,

We appreciate your time and consideration in reviewing our manuscript. We have carefully addressed the comments provided and made the necessary revisions. Below, we outline the specific changes made in response to your feedback:

1. Background

Comment: The background needs rewriting.

Response: We have rewritten the background section. The revised background now provides a concise and coherent overview, moving from a global perspective to Africa, and then specifically to Ethiopia

2. Methodology

Comment: The methodology requires revision.

Response: The methodology section has been extensively revised. We have clarified the sampling technique and included a diagram illustrating the number of participants recruited from each hospital. Additionally, we have provided detailed information on the data collection methods, including the use of both primary and secondary data. Specific details regarding the testing procedures for HIV, HBV, and Syphilis, including the names of the screening kits and confirmation tools and their countries of manufacture, have been added.

3. Conclusion

Comment: The conclusion should be rewritten based on the study's findings.

Response: The conclusion has been rewritten to accurately reflect the study's findings. It now provides a clear summary of the results and their implications, along with recommendations for future research.

4. Ethical Approval

Comment: The authors must also submit a letter of approval from the institutional ethics committee along with the revised manuscript. A supportive letter from the department will not be accepted.

**Response:** In our university, ethical clearance is provided by the school/ department on behalf of the university(the institute). We understand the need for an institutional-level approval and have thus included an approval letter from the department, which serves as the authorized body for ethical review in our institution. We appreciate your understanding of this process and have ensured that all ethical considerations are thoroughly addressed in the like this.

We believe these revisions have significantly strengthened our manuscript and have addressed all the points raised. Thank you for considering our revised submission. We look forward to your favorable response.

Sincerely,

Degsew Ewunetie

Department of clinical midwifery, school of midwifery, College of Medicine and Health Science, Woldia university, Woldia , Ethiopia

E-mail: degsewewunetie@gmail.com

To: The Editors of PLOS ONE

Re: Manuscript PONE-D-24-25429

‘Seroprevalence of HIV, HBV, and syphilis co-infections and its associated factors among pregnant women attending antenatal care in Amhara regional state, northern Ethiopia'

Dear Editors,

We appreciate the reviewers' thorough evaluation of our manuscript and their valuable comments. We have addressed each point raised and made the necessary revisions to improve the quality of our manuscript. Below, we provide a detailed response to each comment:

**Reviewer #1**

1. Abstract: Is the P-value ≤ 0.05 accurate?

- Response: We have double-checked the statistical analyses and confirm that the P-value < 0.05 is accurate. The abstract has been updated for clarity.

2. Background: The background section is overly extensive; the author needs to eliminate redundancies and revise it.

- Response: We have revised the background section to eliminate redundancies and the content for better clarity and focus.

3. Methodology
   - Sampling technique:

Response: We have clarified the sampling technique and included a diagram illustrating the number of participants recruited from each hospital.

- Data Collection Method:

i. Data extraction checklist:

Response: Yes, a data extraction checklist was utilized.

ii. Test results for subsequent visits

Response: We have added an explanation regarding how test results for subsequent visits were obtained. Typically, hospitals conducted tests during the initial visit, and additional tests were performed based on clinical indications and after 37 weeks . the reason for all ANC women included is that the coinfection status of the women can influenced by the number of contact. If the women for instance is infected with syphilis in the first contact another test can she get on subsequent visit.

- iii. Testing procedures:
  - Response: Details of the HIV, HBV, and Syphilis testing procedures employed in the hospitals have been added.
- iv. Screening kits and confirmation tools:
  - Response: The names of the screening kits, confirmation tools, and their countries of manufacture have been specified.

4. Data Quality Control

- Response: We have implemented quality control measures for laboratory tests and quality assurance for the checklist used in chart reviews, which are now detailed in the manuscript.

5. Results

- Response: The sociodemographic factors have been analyzed but no one variables were associated with each co-infection.

6. Discussion

- Response: The discussion section has been condensed to highlight significant implications. Limitations associated with the cross-sectional study design have not been acknowledged because cross-sectional study design is not the limitation of my study .

7. Conclusion

- Response: We have revised the conclusion to provide a more accurate reflection of the findings.

8. References

- Response: DOIs have been included in the references.

**Reviewer #2**

1. Conclusion alignment

- Response: The conclusion has been revised to align more closely with the study's findings.

2. Background restructuring

- Response: The background section has been restructured to flow from a global perspective to Africa, and then specifically to Ethiopia.

3. Inclusion of all ANC attendees

- Response: We have clarified that all ANC attendees (1st, 2nd, 3rd, and 4th visits) were included in the study.

4. Inability to directly test for HIV, HBV, and Syphilis:

- Response: The reasons for the study's reliance on secondary data for HIV, HBV, and Syphilis testing are now clearly explained. because all referral hospitals are done the test routinely for all women. So doing the test again is time and financial waste.

5. Ethical clearance:

- **Response:** In our university, ethical clearance is provided by the school/ department on behalf of the university(the institute). We understand the need for an institutional-level approval and have thus included an approval letter from the department, which serves as the authorized body for ethical review in our institution. We appreciate your understanding of this process and have ensured that all ethical considerations are thoroughly addressed this means. So We have obtained and included an ethical approval letter endorsed by the university/college-level review committee on behalf of the university given by the department. Ethical considerations during the review of test results have also been addressed.

6. Results section

- Response: We appreciate the positive feedback Discussion paragraph removal:

7. Discussion

- Response: The first paragraph of the discussion has been removed as suggested.

8. Study strengths:

- Response: The strengths of our study have been highlighted in the discussion section.

9. High prevalence baseline

- Response: We have included information on the baseline used for comparison in discussing the high prevalence observed.

10. Recommendations for future research:

- Response: Recommendations for future research have been added.

11. Conclusion revision:

- Response: The conclusion has been thoroughly revised to better reflect the study's findings.

12. Duplicated Figure 1

- Response: The duplicated Figure 1 has been removed.

We believe these revisions have significantly improved our manuscript and addressed the reviewers' concerns. Thank you for considering our revised submission. We look forward to your favorable response.

Sincerely,

Degsew Ewunetie,

Department of clinical midwifery, school of midwifery, College of Medicine and Health Science, Woldia university, Woldia , Ethiopia

E-mail: degsewewunetie@gmail.com
