# Supplementary material for: Seroprevalence of HIV, HBV, and syphilis co-infections and associated factors among pregnant women attending antenatal care in Amhara regional state, northern Ethiopia: A hospital-based cross-sectional study
Source: PLoS One. 2024 Aug 8;19(8):e0308634. doi: 10.1371/journal.pone.0308634 (PMC11309376; doi:10.1371/journal.pone.0308634)
Supplement: Attachment — Submitted filename: Response to ReviewersR1.docx [file pone.0308634.s003.docx]

To: The Academic Editor, PLOS ONE

Re: Manuscript PONE-D-24-25429R1

'Seroprevalence of HIV, HBV, and Syphilis Co-infections and Its Associated Factors Among Pregnant Women Attending Antenatal Care in Amhara Regional State, Northern Ethiopia'

Dear Academic Editor,

We appreciate your time and consideration in reviewing our manuscript. We have carefully addressed the comments provided and made the necessary revisions. Below, we outline the specific changes made in response to your feedback:

**1. The manuscript requires further language editing due to numerous instances of poor language**

**Response**: We have thoroughly revised the manuscript to improve the language and readability. We have employed a professional editing service to ensure that the language meets the standards required by PLOS ONE. The revised manuscript should now be free of any language issues.

**2. Please add the study design to the end of your study's title**

**Response**: We have modified the title of our manuscript to include the study design as per the journal's guidelines. The new title is: "Seroprevalence of HIV, HBV, and Syphilis co-infections and its associated factors among pregnant women attending antenatal care in Amhara regional state, Northern Ethiopia: A hospital -based cross-sectional study."

**3. Journal Requirements**

Please review your reference list to ensure that it is complete and correct. If you have cited papers that have been retracted, please include the rationale for doing so in the manuscript text, or remove these references and replace them with relevant current references. Any changes to the reference list should be mentioned in the rebuttal letter that accompanies your revised manuscript. If you need to cite a retracted article, indicate the article’s retracted status in the References list and also include a citation and full reference for the retraction notice

**Response**: We have reviewed our reference list to ensure that it is complete and correct. During this review, we have not identified problems on the listed references. If there we are happy to revise the list again.

We hope that these revisions adequately address the concerns raised and that the revised manuscript meets the publication criteria for PLOS ONE. Thank you for your time and consideration. We look forward to your positive response.

Sincerely,

Degsew Ewunetie

Department of clinical midwifery, school of midwifery, College of Medicine and Health Science, Woldia university, Woldia , Ethiopia

E-mail: [degsewewunetie@gmail.com](mailto:degsewewunetie@gmail.com)
